# The Role of Mechanistic Target of Rapamycin (mTOR) Complexes Signaling in the Immune Responses

**DOI:** 10.3390/nu5062231

**Published:** 2013-06-19

**Authors:** Ghada A. Soliman

**Affiliations:** Department of Health Promotion, Social and Behavioral Health Sciences, College of Public Health, University of Nebraska Medical Center, 984365 Nebraska Medical Center, Omaha, NE 68198, USA; E-Mail: ghada.soliman@unmc.edu; Tel.: +1-402-559-5157; Fax: +1-402-559-3773

**Keywords:** mTOR, innate immunity, adaptive immunity, regulatory T cells, T helper, B cells, rapamycin, immunosuppression, mTORC1, mTORC2

## Abstract

The mechanistic Target of Rapamycin (mTOR) is an evolutionarily conserved serine/threonine kinase which is a member of the PI3K related kinase (PIKK) family. mTOR emerged as a central node in cellular metabolism, cell growth, and differentiation, as well as cancer metabolism. mTOR senses the nutrients, energy, insulin, growth factors, and environmental cues and transmits signals to downstream targets to effectuate the cellular and metabolic response. Recently, mTOR was also implicated in the regulation of both the innate and adaptive immune responses. This paper will summarize the current knowledge of mTOR, as related to the immune microenvironment and immune responses.

## 1. Introduction

The evolutionarily conserved mechanistic Target of Rapamycin (mTOR, formerly known as mammalian TOR) protein kinase plays an integral role in the coordination of metabolism, protein synthesis, cell growth, and proliferation [1,2,3,4,5]. mTOR serine/threonine kinase also functions as a molecular sensor of metabolism and cellular homeostasis and integrates environmental signals by altering the cellular metabolic processes [5,6]. Studies in yeast and *Drosophila* provided significant insight into the Target of Rapamycin (TOR) homolog physiology [7,8,9,10]. TOR was originally identified in *S. cerevisiae* as a mutant that confers rapamycin resistance [11]. Subsequently, TOR signal transduction pathways were elucidated in several organisms and thereafter the mammalian homologs were identified [5,12,13,14].

## 2. mTOR Signaling Pathway

Recent studies have implicated mTOR as a central regulator of metabolism, growth, cellular proliferation, and cell cycle progression [2,15,16]. Further, compelling evidence revealed that the dysregulation of mTOR is linked to the development of chronic diseases including insulin resistance, diabetes, cardiovascular disease, and obesity [17]; as well as progression of various types of cancer [18,19]. More recent work indicates that mTOR Complex 1 (mTORC1) plays a significant role in protein synthesis [2], lipid biosynthesis [15], and inhibition of triacylglycerol lipolysis [20,21]. Thus it may have a significant impact on maintaining metabolic homeostasis in the whole body [3].

### 2.1. Upstream of mTOR

#### mTOR Multiple Signaling Components

**Figure 1:**
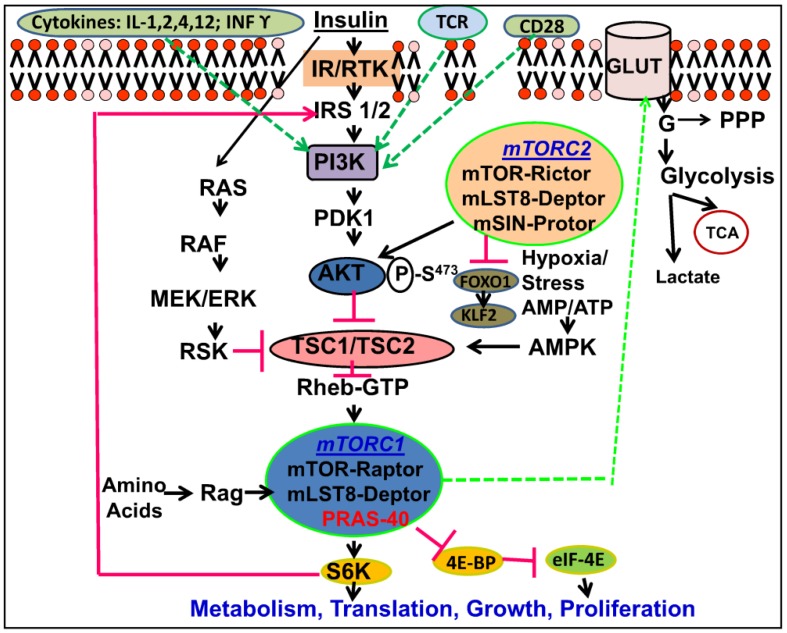
Network of the mechanistic Target of Rapamycin (mTOR) signaling pathway. Figure 1 shows diagram summarizing the inputs and outputs of mTOR signaling network. mTORC1 integrates multiple inputs from signaling pathways including insulin, growth factors, energy, stress, mitogens, and amino acids. Additionally PI3K-mTOR also receives signals from the immune microenvironment such as T Cell Receptors (TCR), and co-stimulatory molecules CD28 (Cluster of Differentiation 28), as well as interleukins. mTORC2 complex activates Akt by phosphorylating it on serine 473, and indirectly activates mTORC1, which in turns phosphorylates its effector S6K on threonine 389.

mTOR governs and integrates signals from multiple signaling pathways including insulin signaling, growth factors, energy, stress, mitogens, and amino acids, particularly the branched chain amino acid (BCAA) leucine (Figure 1). However, the mechanisms by which amino acids activate mTOR pathway are not fully understood. Amino acids activate the Rag GTPases (Rag guanosine triphosphatases), which interact with small complex proteins collectively known as Ragulators which facilitate docking of Rag to the lysosomal surface. This association in turn promotes the localization of mTOR and Rheb (Ras homolog enriched in the brain) to the lysosomes in response to amino acids [22,23]. Recently, it has been shown that PI3K-Akt-mTORC1 axis also receives signals from the immune microenvironment such as the co-stimulatory molecules CD28 (Cluster of Differentiation 28), as well as interleukins including IL-1, IL-2, IL-4, IL-12, and Interferon gamma (IFN-γ) [24]. Immunologically germane cytokines including CD28, and IL-2, IL-4, as well as growth factor receptors have been shown to activate mTOR via PI3 kinase upregulation [25,26]. Additionally, upon activation of mTOR, IL-12 and IFNγ prolong this activation state in memory CD8+ cells [27,28]. In a similar manner, IL-1 has been shown to promote the development of T helper 17 (Th17) via activation of mTORC1 complex [29,30]. Powell and colleagues [24] proposed a model were mTOR integrates signals from metabolic, environmental, and growth factors cues, as well as cytokines and co-stimulatory signals to dictate the outcome of antigen recognition by T Cells following T Cell Receptor engagement. Indeed, validation of this model is currently under investigation. Substantial breakthrough in mTOR research led to the identification of the Tuberous Sclerosis Complex (TSC1 and TSC2) as upstream negative regulators of mTOR [31,32,33,34,35,36] TSC1/TSC2 complex is negatively regulated by the serine/threonine kinase PKB/AKT downstream of the insulin-signaling pathway (Figure 1) and has GTPase-activating domain (GAP) in the *C*-terminus. Recent studies [37,38] showed that Rheb, a Ras family small GTPase, is a direct target of TSC2’s intrinsic GTPAse activity (Figure 1). Furthermore, an attractive model revealed that TSC-2 is a direct substrate of AMPK (AMP activated protein kinase), which serves as an intracellular sensor of the AMP/ATP ratio both *in vivo* and *in vitro* [36]. As such, when the energy level is low, AMPK phosphorylates and activates TSC2, which inactivates Rheb.GTP and converts it to Rheb.GDP, and in turn this leads to mTOR inhibition. The discovery of multiple upstream regulators of mTOR, together with the documented negative feedback loop from S6 Kinase 1 (S6K1) on Insulin Receptor Substrate 1 (IRS) [39], increased our understanding of mTOR signaling pathways and placed mTOR downstream of the linear insulin signaling cascade (Figure 1).

**Figure 2:**
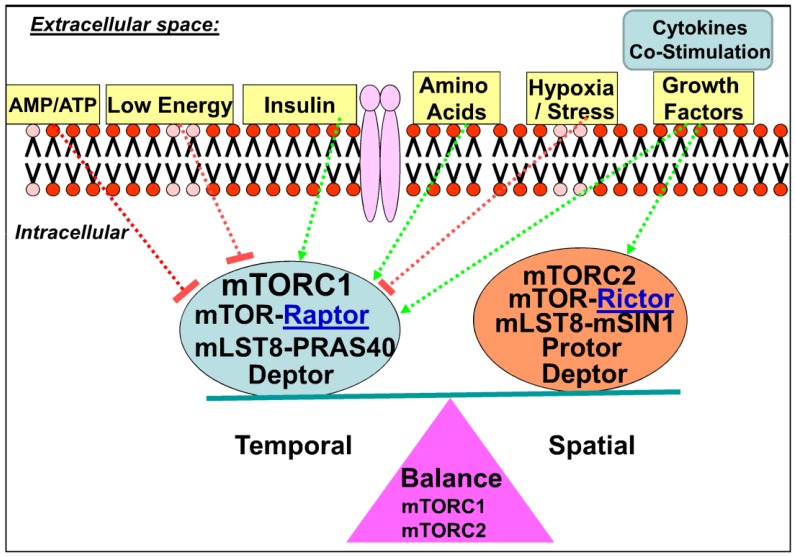
mTORC1 and mTORC2 integrate signals from diverse extracellular inputs. mTOR complexes integrate signals from diverse environmental cues. mTOR forms at least two complexes with the newly identified exclusive as well as common binding partners. These complexes include rapamycin-sensitive complex 1 (mTORC1) encompassing mTOR, raptor, mLST8, PRAS40, and deptor; and rapamycin-insensitive complex 2 (mTORC2), which contains mTOR, rictor, mLST8, mSIN1, protor, and deptor.

Furthermore, mTOR forms at least two complexes with the newly identified exclusive as well as common binding partners as shown below (Figure 2). These complexes include rapamycin-sensitive complex (mTORC1) encompassing mTOR, raptor, mLST8, PRAS40, and deptor and rapamycin-insensitive complex (mTORC2), which contains mTOR, rictor, mLST8, mSIN1, protor, and deptor (but not raptor).

### 2.2. mTOR Complexes

Genetic studies in mice yielded significant understanding of the components of mTOR complexes as well as its binding partners, upstream effectors, and downstream molecular targets [2]. mTOR is the catalytic core of two distinct multi-protein complexes, mTOR Complex 1 (mTORC1) and mTOR Complex 2 (mTORC2) as shown in Figure 2. mTORC1 comprises mTOR kinase, mTOR-raptor binding partner (raptor), and mLST8, DEPTOR, and PRAS 40; and integrates signals from nutrients, insulin, growth factors, and energy levels, thereby activates downstream targets to control metabolism and cell growth [40,41].While mTORC1 is a nutrient-sensitive pathway, mTORC2 receives inputs only from growth factors, however mTORC2 upstream regulation is largely unknown [42].

Deletion of rictor, the rapamycin-insensitive mTORC2, which phosphorylates AKT-Ser^473^ [43] led to glucose intolerance due to discordant beta cell proliferation and cell size. Aberrant activation of mTORC1 has been implicated in a variety of pathological conditions including obesity, diabetes, and cancer [3,4,44]. Furthermore, the hallmark of cancer is elevated glucose consumption, increased glycolysis, and lactate productions commonly known as oxidative glycolysis or Warburg effect [45,46]. A similar oxidative glycolysis pathway is also utilized by the lymphocytes to generate energy in the form of ATP. Thus cancer metabolism and T-lymphocyte metabolism share many similarities in energy metabolism and utilization. Although lactate utilization is an inefficient way to generate energy, the oxidative glycolysis pathway allows for the accumulation of substrates and byproducts needed for the biosynthesis. Thus the immunological functions of T cells can be regulated by the specialized metabolic program [24]. Failure to upregulate the metabolic machinery upon immunologic rechallenges is currently thought to lead to the hypo-responsiveness status of T cells known as anergy [47,48]. This anergy status will ultimately lead to immunologic tolerance. Indeed, it has been postulated that rapamycin inhibits T cell proliferation and causes immunosuppression due to its ability to promote T Cell anergy [25,49,50,51].

### 2.3. Downstream of mTOR

It is currently known that mTORC1 phosphorylates two well characterized downstream targets, S6K1 and 4E-BP1 (eIF-4E binding protein), positive and negative regulators of protein synthesis, respectively (Figure 1). Phosphorylation of S6K1, in turn, phosphorylates and thereby activates the downstream 40S ribosomal protein S6 and enhances translation of mRNA (Figure 1). On the contrary, phosphorylation of 4E-BP1 induces its release from eIF4E, and as such, enhances eIF4E-mediated cap dependent translation [52,53,54]. mTOR-dependent signaling, along with both S6K1 and 4E-BP1 pathways, independently drive cell growth (cell mass and size) and proliferation [55,56]. Additionally, mTORC2 activation inhibits two proteins namely, Forkhead Factor 1 (FOXO1) and Kruppell-like factor 2 (KLP), two transcription factors required for maintaining the T cell quiescent state [57,58,59]. Thus, it appears that both mTORC1 and mTORC2 pathways play a role during the immune response.

### 2.4. mTOR Pathways and Metabolism

#### 2.4.1. mTOR Regulation of Anabolic and Catabolic Pathways

mTOR, the downstream target of insulin action (Figure 1), influences the regulation of gene transcription [60,61] as well as protein translation [52,62,63,64], and cell signaling [65,66]. As stated earlier, in yeast, TOR has been shown to serve as a master switch between protein synthesis and anabolism on one hand, and autophagy, which is indicative of a catabolic state on the other hand [65]. The mammalian homolog, mTOR has emerged as a key integrator of both *anabolic* and *catabolic* processes [2,15,16]. The anabolic role of mTOR is evident in the regulation of lipogenesis as mTORC1 activates the transcription factor SREBP-1, a major transcription factor that controls fatty acid, cholesterol, and triglyceride synthesis [67,68,69], as well as activation of Stearoyl CoA desaturase (SCD1), a key enzyme in fatty acid metabolism required for double bond formation [70]. The inhibition of triacylglycerol (TAG) lipolysis suggests the importance of mTOR in blocking catabolic pathways [39,71]. mTORC1 activation suppresses the nutrient-recycling process known as autophagy. In such process, cellular organelles and proteins are degraded by lysosomes and the components are recycled and utilized for energy production [72,73].

#### 2.4.2. mTOR and Carbohydrate Metabolism

Glucose homeostasis is orchestrated by interactions of the two pancreatic hormones namely, insulin and glucagon. Insulin promotes glycogen storage and glucose utilization by regulating both gene transcription [65,66] as well as cell signaling [74]. The dual impact of the insulin downstream target, mTOR on the regulation of gene transcription [60,61], as well as protein translation and cell signaling [52,62,63,64], have also been documented. mTORC1 nutrient sensing pathway responds to glucose and insulin that controls diverse cellular processes including protein synthesis, cell metabolism, and cell growth. However, the biochemical link between mTORC1 signal transduction and metabolic homeostasis remains unclear [44]. mTORC1 enhances the translation of Hypoxia-inducible factor 1α (HIF1α), a transcription factor, that in turn regulates the transcription of genes encoding glycolytic enzymes and glucose transporters [75,76,77]. As a result, mTORC1 promotes glucose uptake and activation of glycolysis to generate energy. Recently, it has been shown that mTOR interacts with glycogen synthase kinase (GSK-3) to increase DNA synthesis [78] and regulates glucose-6-phosphate dehydrogenase (G6PDH) activity [16,79]. Additionally, there is cross talk between mTOR and G6PDH [16,79], and AMPK [80,81]. G6PDH catalyzes the irreversible oxidation of G6P to 6-phosphogluconolactone, the rate-limiting reaction in the pentose phosphate pathway (PPP), which generates ribose for RNA, and DNA synthesis as well as NADPH for fatty acid synthesis. mTORC1 is also known to increase cell growth and proliferation by activation of cell cycle progression [82]. Thus, mTORC1 may influence cell proliferation through dual mechanisms: (1) cell cycle progression, and (2) metabolic channeling of glucose through the pentose phosphate pathway. Thus, mTOR serves as a central integrator of growth and metabolism [5].

#### 2.4.3. mTOR and Lipid Metabolism

As mentioned earlier, emerging data identify mTORC1 as an important novel controller of both anabolic and catabolic lipid metabolism by regulating lipogenesis and lipolysis, respectively [20,69,83,84,85]. mTORC1 signaling induces adipogenic differentiation and maintains the adipogenic program by promoting the expression and the activation state of transcription factor PPARγ (peroxisome proliferator-activated receptor-γ), a nuclear hormone receptor that induces the expression of genes which promote fatty acid uptake, synthesis, esterification, and storage [50]. Moreover, mTORC1 signaling promotes lipid biosynthesis by cleaving the transcription factor SREBP-1 (sterol regulatory element binding protein-1) into a mature form that translocates to the nucleus to induce the expression of lipogenic genes that promote fatty acid and TAG synthesis [15,84]. Additionally, two independent laboratories showed that inhibition of mTORC1 signaling via rapamycin or knockdown of the critical mTORC1 partner raptor decreases triacylglycerol (TAG) storage by increasing lipolysis in 3T3-L1 adipocytes [21], and increase plasma FFA in guinea pigs [20]. It is well known that increased levels of circulating FFA is a risk factor in the development of insulin resistance, the landmark of type 2 diabetes. Furthermore, cumulative data suggest that rapamycin administration to patients may exacerbate insulin resistance by promoting TAG lipolysis [86,87].

#### 2.4.4. mTOR and Protein Metabolism

Recent studies indicate that mTOR mediates nutritional signaling checkpoints to diverse nutritional clues [88,89,90]. Furthermore, rapamycin treatment leads to a starvation-like response; suggesting the biological role of mTOR in integrating nutritional signals [91]. During starvation, the cell will recycle its organelles by autophagy to provide substrates for energy production. This will provide a source of amino acids that will ultimately activate mTOR to generate energy. In yeast, TOR serves as an integrative regulator of genes required for *de novo* biosynthesis of glutamine and glutamate [92]. Moreover, branched chain amino acids, particularly leucine, have been shown to stimulate mTOR signaling and enhance phosphorylation of 4E-BP and ribosomal protein 6 kinase [93,94]. During nutrient deprivation, stress, or mTOR inhibition by rapamycin, autophagy is induced. Likewise, the lymphocytes are catabolic during the resting state, and thus they employ autophagy to provide molecules required for energy production [95]. mTOR central role in regulating autophagy and metabolic programs provides a pivotal link between cellular metabolism and the innate as well as the adaptive immune response.

## 3. mTOR and the Innate Immunity

The innate immune system is an immediate protective response with broad activity upon entry of microorganisms. The innate immunity relies on mechanisms that are established prior to infection and are employed to rapidly attack the offending microbes. Components of the innate system facilitate phagocytosis and lysis of bacteria by virtue of epithelial barriers; mucosal membranes; phagocytes (monocytes, macrophages, and neutrophils); complements, natural killer cells (NK); cytokines produced by the phagocytes; and dendritic cells [96]. These bone-marrow derived dendritic cells (DC) with multiple long membranous projections share common progenitors with macrophages, and thus share some overlapping functions such as phagocytosis [96]. Dendritic cells function as Antigen Presenting Cells (APC) to activate naïve T cells. Once activated, the naïve T cells initiate the adaptive immune response. Powell and colleagues propose that mTOR senses the immune microenvironment and directs the outcome of antigen recognition, in a manner similar to mTOR-mediated nutrient-sensing, and in response to energy and environmental cues [24]. The group suggests that mTOR derives the differentiation and function of APC. Indeed, mTOR has been shown to sense signals from the dendritic cells, which function as antigen presenting cells to the naïve T lymphocytes in order to; direct T-cell activation, differentiation, and clonal expansion [97,98]. By presenting antigens to T-cells, Dendritic Cells (DCs) serve as conduit and link between innate and adaptive immunity. As such, DCs migrate to the tissues, sample their environment and act as sentinels for signs of infection. The DCs are equipped with Toll-like Receptors (TLR), and Pattern Recognition Receptors (PRR). The PRR identifies the Pathogen Associated Molecular Pattern (PAMPs) and presents the antigens to the T cells of the adaptive immune response. Dendritic cells secret pro-inflammatory cytokines and serve as Antigen Presenting Cells (APC) to present foreign antigens to activate the CD4+ T cells.

The effect of mTOR inhibition by rapamycin treatment varies depending on the type of DC cells activated [99], and also whether rapamycin was used on short-term or long-term bases [100]. These factors play an important role on the diverse effect of mTOR inhibition during DC cells activation and differentiation [99,101,102]. As such, short-term treatment with rapamycin has been shown to increase IL-12, IL-1β cytokine production and NF-κB in TLR-2 activated monocyte-derived DC and in myeloid-derived DCs [99]. However, long-term treatment with rapamycin decreases the innate immunity in monocyte-derived DC cells [103,104,105,106]. Rapamycin treatment also abolished the ability of the Toll-Like Receptors (TLR) TLR 7 and TLR 9 to induce IFN α and IFN β required for the antiviral immune response. Furthermore, rapamycin also decreases the development of dendritic cells including both the conventional and the plasmacyoidal DC downstream of cytokine Flt3 ligand (Flt3L) *in vitro* [104,107]. Rapamycin also reduces the dendritic cell co-stimulatory molecules upregulation [104]. In addition, rapamycin treatment was associated with impaired dendritic cells functions such as reduced allogeneic T cell responses [108], while it increased the regulatory T antigen specific Foxp3+ [108]. It is worth noting that renal transplant patients treated with rapamycin exhibited an increase in the immunostimulatory potential of myeloid-derived DC compared to patients treated with cyclosporin or FK506 [99].

Importantly, rapamycin has been shown to suppress the mature dendritic cell function and decrease the production of cytokine IFNγ produced by dendritic cells, the cytokine that represents the first defense against viral infection [99]. Further, knockout of raptor, an integral component of mTORC1 component reveals a phenotype of expanded splenic CD8+, and intestinal CD11+, indicating that mTORC1 plays a significant anti-inflammatory role in intestinal infection [109], and maintains intestinal homeostasis by promoting the anti-inflammatory cytokine, IL-10 [109].

**Figure 3:**
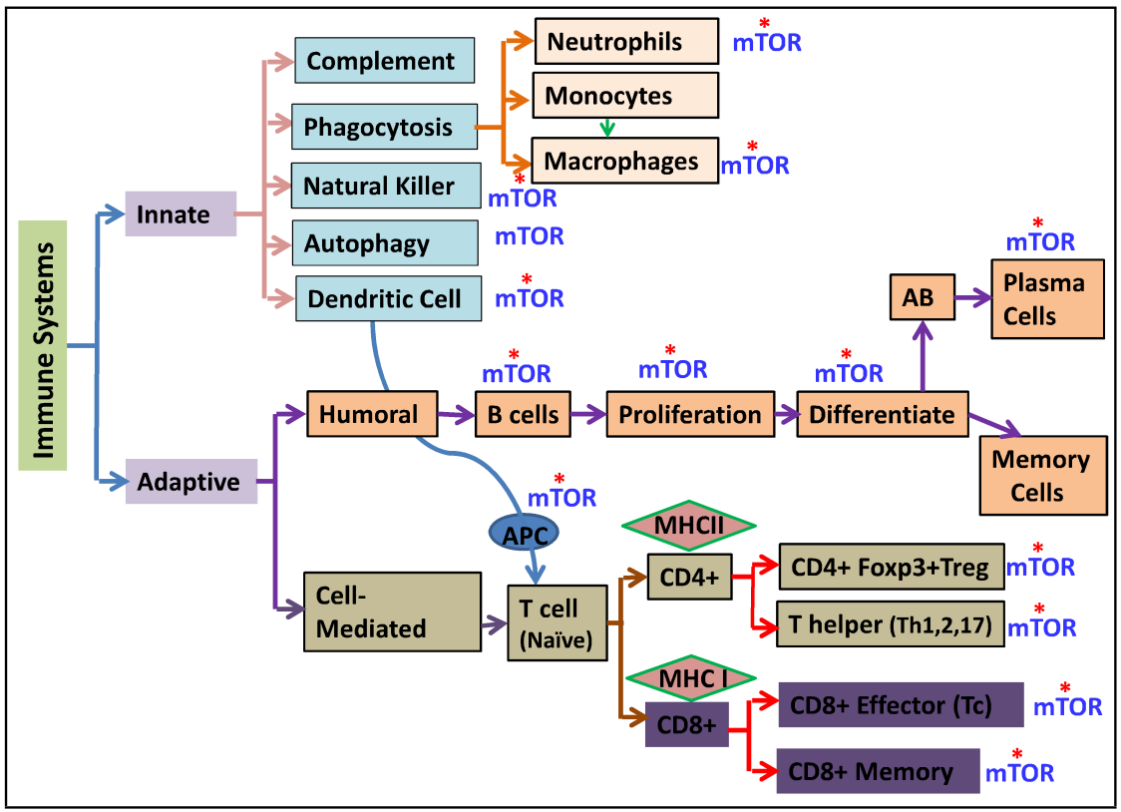
Reported sites of actions of mTOR on the immune response. Figure 3 summarizes an overview of the reported sites action of mTOR interactions with the immune system. mTOR has been shown to affect multiple components of the immune response as shown by asterisk (*) on the diagram. This encompasses both the innate and adaptive immune system. While the innate immune response is immediate and board in scope, the adaptive immune response is delayed and highly specific in function.

Furthermore, mTOR also plays an important role in regulation of other components of the innate immunity [110] including Natural Killer (NK) cells in kidney transplant patients [111], neutrophils, and macrophages activities [48,112,113,114,115,116] as shown in Figure 3. In humans, rapamycin treated renal transplant patients show a prostimulatory pattern of myeloid dendritic cells [99]. mTOR inhibition also is shown to enhance proinflammatory cytokines, including IL-12 and IL-1β [102]. Although rapamycin is clinically administered as immunosuppressant, these observations suggest the presence of distinct inflammatory side effects of rapamycin [99,102]. However, the significance and clinical implications of these observations remain to be elucidated.

## 4. mTOR and Adaptive Immunity

Unlike the innate immune response, the adaptive immune response is delayed but is highly specific to the nature of infection. The adaptive response encompasses, the humoral response mediated by B cells and the cell-mediated response triggered by T lymphocytes. This immune-specific response improves every time the organism is encountered due to the developing an immune memory for that particular organism.

### 4.1. mTOR and Humoral Immunity (B Cells)

B lymphocytes arise from the bone marrow to produce antibodies (Ab) and, thus, are integral components of the humoral immunity. The humoral response component of the adaptive immunity recognizes the antigens while they are in the blood stream and mucosal surfaces, and before the organism invades the cells and replicates intracellularly. *In vitro* mitogen activated cell culture studies have shown that rapamycin reduces B lymphocyte proliferation and differentiation to plasma cells [117,118,119,120,121].

In a hypomorphic mouse model due to partial disruption of mTOR transcript, Zhang *et al.* reported that these mice exhibited reduced B-cell development, decreased cell proliferation in response to B cell mitogens, and reduced both T-cell dependent and T cell-independent antibody formation [122]. On the contrary, deletion of TSC1/TCS2 complex, a negative regulator of mTOR led to decrease in the Marginal Zone (MZ) of B cells development and significant reduction in B maturation. This effect was partially reversed by rapamycin [123]. The apparent contradictory findings [122] may suggest that mTOR effects on B cell are dictated in part by the relative contribution and ratio between mTORC1 and mTORC2. In line of this argument, Llorian and colleagues [124], reported that mTORC2 plays a critical role in B cell development via activation of AKT phosphorylation at serine 473, and inhibition of FOXO1 signaling.

During prolonged stimulation, B Cell Receptor (BCR) has been shown to increase mTOR activation. However, with minimal antigen exposure, there is limited magnitude of mTOR activation [121,125]. These observations suggest that mTOR plays a critical role in integrating the inputs that affect B cell functions and is regulated by the level of antigen titration. In addition, studies revealed that rapamycin treatment inhibits LPS-induced B cell proliferation and differentiation [121,126]. Along these lines, genetic studies in mouse models showed that mTOR is necessary and required for B cell differentiation to plasma cells [122]. Goldfinger *et al*. reported that within plasma cells which secret abundant level of immunoglobulins, the protein synthesis was regulated by coordination and crosstalk between mTOR and ER stress [127]. Taken together, these observations indicate the mTOR plays a significant role in B cell maturation and differentiation.

### 4.2. mTOR and Cell-Mediated Immunity

While it was originally thought that the T cells were activated upon antigen recognition by T-cell receptors (TCR), as well as co-stimulation and cytokine secretion; it is now recognized that environmental cues play an integral role in T cell activation outcome [128]. These factors include glucose, amino acids, AMP/ATP, and stress signal via activation of the mTOR pathway (Figure 1), which in turn determines the fate of activated T cells. This new concept links, once again, the metabolic nutrient cues to the development of the immune responses.

#### 4.2.1. mTOR and Antigen Presenting Cells

As mentioned earlier, dendritic cells transduce signals received from antigens, co-stimulatory molecules, and pro-inflammatory molecules, which inform the naïve T cells to become activated and undergo clonal expansion and differentiation to become effector T cells. The magnitude of T cell activation *versus* tolerance is dependent on signaling from mTOR as well as NF-κB pathways [129]. Dendritic T cells are equipped with PRR and TLR and serve as sentinels to monitor microbial infection and present the antigen to the T-cell for further processing. In doing so, the dendritic cells call upon membrane complex termed the Major Histocompatibility (MHC) molecule. The MHC serves as the platform to carry the antigen to naïve T-cells. It is worth noting that a role of mTOR in the uptake and processing antigens by the bone-marrow-derived dendritic cells has been reported by Hachstein and colleagues [104,105,130]. Furthermore, the process of dendritic cell maturation has also been documented to be mTORC1 dependent. In this regard, targeted-deletion of mTOR negative regulator, PTEN inhibited the FGFR1 (Fibroblast Growth Factor Receptor, also known as FLT2) ligand-induced DC maturation [107]. As a result, hyperactivation of mTOR signaling led to excessive expansion of DC subset composition *in vivo* [107], and rapamycin inhibited such effect.

Adding to the complexity of mTOR signaling, is the co-stimulatory input received from CD28 which activates PI3K/AKT. The co-stimulatory CD28 input in turn, upregulates the TCR-mediated mTOR activation in order to facilitate T cell clonal expansion [25]. The CD28 is a homodimer chain present on all CD4+ T cells and serves as T cell receptor for co-stimulatory molecules. The CD4 is a co-receptor for MHC class II expressed by T cells and is required-together with MHC class II-for T cell interactions with TCR. Several investigators documented that mTOR activation enables IL-2 independent T cell proliferation [25]. However, in the antigen-stimulated CD8+ T cells, IL-12 induced mTOR activation appears to be indirect via activation of STAT4 signaling [27]. mTOR has been reported to serve as a controller to balance between naïve CD8+ cells differentiation to effector cells versus memory cell formation [28]. This balance dictates whether an immune response is achieved by the CD8+ effector cells or tolerance and hyporesponesiveness is generated by T cell anergy. Furthermore, increased mTORC1 activity via loss of TSC complex mediated-inhibition, has been shown to decrease interleukin 7 (IL-7)-dependent responses to naïve T cells, and to disrupt the immune homeostasis mediated by TSC 1/2 [131]. Along the same line, Zhang *et al*. reported that TSC 1/2 also mediates CD8+ naïve T Cell homeostasis and survival in mice via Akt-mTOR-FOXO pathway [132].

#### 4.2.2. mTOR and T Cell Functions

mTOR emerged as a central node in the control of cellular metabolism. Recently, it has been reported that mTOR integrates cues from the immune microenvironment and drives T-Cell differentiation and function [48]. As mentioned earlier, mTOR has been shown to regulate cycle cell progression and cell growth and proliferation [133,134]. During the adaptive immune response, mTOR signaling determines T cell fate decisions whether towards T cell differentiation or generation of T helper cells. The metabolic program of T cells is very energetically demanding [135], and exhibits preference to aerobic glycolysis, thus mimicking cancer cell metabolism (Warburg Effect). This reprograming of resting T Cells requires receptor mediated signal transduction [135]. Recently, Rosborough *et al*., reported that in the dendritic cells, rapamycin-sensitive mTORC1 promotes effector T cell expansion, while rapamycin-insensitive mTORC1 restrains the regulatory T (Treg) induction [114].

#### 4.2.3. mTOR and CD4+ CD25+ FoxP3 Regulatory T Cells (Treg)

The thymus-derived, naturally-occurring CD4+, CD25+ Forkhead Box P3+ (FoxP3+) Regulatory T cells (Treg) suppresses innate and other adaptive immune responses. Tregs are recently known to express FoxP3 (forkhead box P3), an essential Transcription Factor for immune homeostasis and for prevention of autoimmunity [136,137]. Several studies elucidated that Treg has a greater mTOR activity than the conventional T cells, and thus mTOR may play a significant role in regulatory T cell functions. Rapamycin treatment for a prolonged period has been shown to stimulate the generation to regulatory T cells [138,139,140,141,142,143]. Recent reports indicate that simultaneous inhibition of both mTORC1 and mTORC2 signaling pathways are required for activation of FoxP3+T regulatory cells under activating conditions [144]. Intriguingly, it has recently been shown that Hypoxia Inducible Factor 1 (HIF1) enhanced FoxP3+ Treg generation via a mechanism that involved mTOR signaling [145]. Rapamycin was also shown to promote and expand Foxp3+ T regulatory cells [138,141,146]. Intriguingly, Cobbold and colleagues have shown that Treg stimulates tolerance by depleting the BCAA, which are known activators of the mTORC1 signaling [147]. The depletion of essential amino acids, in turn, leads to inhibition of mTOR signaling, and activation the FoxP3 expression, which converts more naïve T cells to regulatory T cells [147,148]. On the other hand, mTORC2 is a negative regulator of transcription factor FOXO1 and FOXO3a, and these transcription factors promote FoxP3 transcription [149,150]. Thus, mTORC2 inhibition in turns promotes Fox3P3 expression and leads to T regulatory expansion. Taken together, it appears that both mTORC1 and mTORC2 complexes have been shown to serve as negative regulator of Treg lineage commitment [151].While on the other hand, mTOR activation antagonizes regulatory T cells and enhances the development of T helper cells [24,152].

#### 4.2.4. mTOR and Differentiation of T Helper Cells (T Cell Activation and T Cell Differentiation)

Studies in mTOR-null T cells revealed that mTOR plays a fundamental role in integrating signals that facilitate T helper (Th) cell differentiation [144,151]. Similarly, when Rheb, a positive upstream regulator of mTORC1, was deleted in T cells, these cells failed to differentiate into Th1, and Th17, but retained their ability to differentiate to Th2 [144]. Furthermore, Rheb (-/-) T cells responded to cytokine IL-4 release by activating the STAT 6 signaling, confirming their ability to differentiate into Th2 cells [144]. Recent studies also revealed that deletion of Rictor, an integral component of mTORC2 inhibits differentiation of CD4+ T cells to both Th1 and Th2 [153,154]. Along the same line, mTORC2 has been shown to activate PKC theta to promote Th2 differentiation [26,153]. Similarly, Delgoffe and colleagues showed a decrease in Th2 activities in Rictor null cells as evidenced by reduction in cytokine IL4-induced activation of Stat 6 signaling [144]. Taken together, these observations [144,153] suggest a differential regulation of mTORC1 and mTORC2 on T cell differentiation into Th1, Th2, and Th17, as well as T helper functions.

#### 4.2.5. mTOR and Regulation of CD8+ T Effector Cells and CD8+ Memory Cells

It is currently thought that mTOR plays an important role in reversing the quiescent state of CD8+ T cells by inhibition of Transcription Factors, ELF4, and KLF4 that function to induce quiescence in the naïve CD8+ T cells [155]. Paradoxically, recent studies have shown that mTOR inhibition by rapamycin leads to immunostimuation of the long-lived memory CD8+ T cells [26,156]. This effect is attributable, in part, to enhancing the cytokine production by macrophages and dendritic cells, and improving antigen presentation [156,157]. In addition, mTORC1 has been shown to regulate the transcriptional programs that determine the fate of CD8+ Cytolytic Cells (CTL) [158,159,160]. Intriguingly, generation of hyperactive mTORC1 in T cells by selective deletion of TSC2, an upstream mTOR inhibitor, has been shown to increase the effector CD8+ activity [24,128,152]. Indeed, activated CD8+ cells can switch between catabolism and anabolism. Thus, it is tempting to speculate that mTOR mediates such effect; however, this thesis remains to be validated. In addition, PI3K/mTORC1 pathway regulates the expression of Hypoxia inducible factor 1 (HIF1) transcriptional factor complex. This mTORC-1 mediated activation is currently thought to be required for glycolysis, glycolytic enzymes activity, and glucose homeostasis in immune-activated CD8+ Cytolytic T cells [160]. Indeed, it has been demonstrated that mTORC1 stabilizes HIF1 in Th17 cells [161,162,163]. It is also noted that during low oxygen conditions, HIF1 activates TSC1/TSC2 complex, which is a negative regulator of mTORC1 [164,165]. Thus, the oxygen status determines whether HIF1 will be activated e.g., during hypoxia conditions, or becomes targeted to ubiquitin degradation.

Upon antigen encounter, trafficking of naïve T cells through the lymphoid tissue is facilitated by chemokine receptor CCR7, and by CD62L cell surface receptors. Once the CD8+ T cell recognizes their cognate antigen, these cells activate mTOR pathway to switch to the anabolic state [159]. The switch between the CD8+ effector cells and CD8+ memory cells is associated with metabolic switch from anabolism to catabolism. Several investigators have implicated mTOR in the regulation of the effector-to-memory ratio and transition between both states [27,28,157,166,167,168,169,170,171,172,173]. As such, low dose rapamycin was shown to promote CD8+ memory T cell production [166,167,168]. Remarkably, the CD8+ rapamycin-stimulated-memory cells exhibited vigorous anti-tumor immunity when rechallenged [27]. Collectively, these studies support the notion that metabolic programs regulate memory cell development and have significant implications on anticancer therapy and vaccine development.

#### 4.2.6. mTOR and T Cell Anergy, Tolerance and Hyporesponsiveness

Several investigators reported that rapamycin treatment induces anergy even in the presence of costimulators [25,49,50,51]. Indeed, Zhang *et al.* reported that mTOR inhibition by rapamycin induces T cell anergy, which is a state of hyporesponsiveness and tolerance in T cells *in vitro* [174]. In this model, specific CD4+ Th1 clone (E7) proliferation was inhibited by rapamycin. However, during the induction phase, treatment with rapamycin blocked the cell cycle and led to T cell anergy. Intriguingly, the state of anergy was reversed by treatment with the pro-inflammatory cytokine IL-2. Likewise, T cells matured in the presence of rapamcyin revealed a phenotype of anergic effector T cells and promoted the generation of regulatory T cells. This dual impact on bone-marrow derived dendritic cells drives the T cell tolerance state [175]. This observation indicates that rapamycin treatment can decrease allograft rejection by promoting T cell tolerance. Indeed, mouse models of solid organ transplantation revealed that rapamycin-derived allogeneic DC induces tolerance that led to reduction of organ rejection [175,176]. Additionally, monocyte-derived DC was also found to be mTOR-dependent [99], and that treatment of these cells with rapamycin led to apopotosis, and generation of tolerogenic dendritic cells. Taken together, these studies indicated that rapamycin can promote effector cell anergy and promote T cell tolerance by inhibiting mTORC1 signaling.

## 5. Pharmacological Inhibition of mTOR

Rapamycin, an mTOR inhibitor, is a clinically approved drug used as an immunosuppressive agent that reduces organ transplant rejection, as well as a cardiology drug that decreases intimal hyperplasia associated with coronary stent restenosis [177,178,179,180,181]. Rapamycin has several clinical applications as an immunosuppressive, antiproliferative, and antitumor agent. However, the wide range of clinical applications is also accompanied by adverse effects, including hyperlipidemia [182,183,184,185]. Rapamycin has been shown to inhibit T cell proliferation and cell cycle progression [55]. In addition, recent evidences in the past few years reveal that mTOR plays a significant role in both the innate and adaptive immune responses [113,186].

Rapamycin binds to immunophilin FKBP12 and forms a ternary complex by binding to the rapamycin binding domain (FRB) on mTOR thereby blocking mTOR functions. Unlike cyclosporine A, rapamycin does not inhibit calcineurin and thus does not inhibit T Cell Receptor-induced NF-AT activation. An advantage of mTOR inhibitors is that, unlike the immunosuppressant calcinuerin inhibitors, rapalogs are antitumorigenic and thus are beneficial in renal transplant as post-transplant malignancy is a serious complication [17,179]. However, several side effects of rapamycin treatment have been reported including hyperlipidemia and delayed wound healing [187,188]. Rapamycin acutely inhibits signaling by mTORC1; however, chronic high doses of rapamycin also inhibit mTORC2 in certain cells, an effect that may be attributable to obstructing mTORC2 assembly [2,189,190,191]. Rapamycin (also known as Sirolimus), and rapalogs (Everolimus, Deforolimus) are currently assessed for cancer treatment in clinical trials phase I [192], II [193], and III [194,195]. However, the results vary across the cancer spectrum, attributable, in part, to the discovery of rapamycin-resistant functions of mTOR, the unfolding intricacies of mTOR complexes, and negative [39] and positive feedbacks [196] on insulin signaling pathway, as well as cross talk with oncogenic and tumor suppressors pathways [3,4]. Intriguingly, Everolimus (RAD001), showed promising results in the treatment of pancreatic neuroendocrine tumors, and thus its use was granted by FDA in 2011 [195].

## 6. Conclusions

The impact of mTOR on cellular metabolism, the immune microenvironment, cell proliferation and differentiation, and cancer metabolism provides an attractive therapeutic target for metabolic diseases as well as cancer early detection and therapeutic interventions.
